# *Ex vivo* characterization of acute myeloid leukemia patients undergoing hypomethylating agents and venetoclax regimen reveals a venetoclax-specific effect on non-suppressive regulatory T cells and *bona fide* PD-1^+^TIM3^+^ exhausted CD8^+^ T cells

**DOI:** 10.3389/fimmu.2024.1386517

**Published:** 2024-05-15

**Authors:** Giulia Corradi, Dorian Forte, Gianluca Cristiano, Andrea Polimeno, Marilena Ciciarello, Valentina Salvestrini, Lorenza Bandini, Valentina Robustelli, Emanuela Ottaviani, Michele Cavo, Darina Ocadlikova, Antonio Curti

**Affiliations:** ^1^ Istituto di Ricovero e Cura a Carattere Scientifico (IRCCS) Azienda Ospedaliero-Universitaria di Bologna, Istituto di Ematologia “Seràgnoli”, Bologna, Italy; ^2^ Department of Oncology Hematology, Pescara Hospital, Pescara, Italy; ^3^ Department of Medical and Surgical Sciences (DIMEC), University of Bologna, Bologna, Italy; ^4^ Consiglio Nazionale delle Ricerche (CNR) Institute of Molecular Genetics “Luigi Luca Cavalli-Sforza”, Unit of Bologna, Bologna, Italy; ^5^ Istituto di Ricovero e Cura a Carattere Scientifico (IRCCS) Istituto Ortopedico Rizzoli, Bologna, Italy

**Keywords:** acute myeloid leukemia, immune system, venetoclax, hypomethylating agents, immune checkpoints receptors, immune checkpoint inhibitors (ICIs)

## Abstract

Acute myeloid leukemia (AML) is an aggressive heterogeneous disease characterized by several alterations of the immune system prompting disease progression and treatment response. The therapies available for AML can affect lymphocyte function, limiting the efficacy of immunotherapy while hindering leukemia-specific immune reactions. Recently, the treatment based on Venetoclax (VEN), a specific B-cell lymphoma 2 (BCL-2) inhibitor, in combination with hypomethylating agents (HMAs) or low-dose cytarabine, has emerged as a promising clinical strategy in AML. To better understand the immunological effect of VEN treatment, we characterized the phenotype and immune checkpoint (IC) receptors’ expression on CD4^+^ and CD8^+^ T cells from AML patients after the first and second cycle of HMA in combination with VEN. HMA and VEN treatment significantly increased the percentage of naïve CD8^+^ T cells and TIM-3^+^ CD4^+^ and CD8^+^ T cells and reduced cytokine-secreting non-suppressive T regulatory cells (Tregs). Of note, a comparison between AML patients treated with HMA only and HMA in combination with VEN revealed the specific contribution of VEN in modulating the immune cell repertoire. Indeed, the reduction of cytokine-secreting non-suppressive Tregs, the increased TIM-3 expression on CD8^+^ T cells, and the reduced co-expression of PD-1 and TIM-3 on both CD4^+^ and CD8^+^ T cells are all VEN-specific. Collectively, our study shed light on immune modulation induced by VEN treatment, providing the rationale for a novel therapeutic combination of VEN and IC inhibitors in AML patients.

## Introduction

Acute myeloid leukemia (AML) is an aggressive heterogeneous disease. Despite recent advances, the 5-year AML patients’ overall survival (OS) is still largely unsatisfactory, reaching only 30% and dropping to 5-10% in the elderly (1). To improve AML patients’ clinical outcomes, novel drugs have entered the clinical stage with favorable results (2). Although their direct mechanisms of action are defined, some bystander and/or off-target effects are not fully elucidated. In particular, the activity of novel drugs in modulating the immune cell repertoire has been poorly investigated.

The immune microenvironment of AML is characterized by a broad spectrum of alterations, including a marked dysfunction in the T cell compartment (3–7), leading to the creation of an immunosuppressive milieu, which favors immune escape and alters the response to therapy (8, 9). Furthermore, AML cells can enhance T cell dysfunction through the expression of ligands activating immune checkpoint (IC) receptors expressed on T cells (9, 10), modulating their functionality (11). These findings have provided the rationale for IC inhibitor (ICI)-based therapies in AML patients. However, despite the solid preclinical background, the early clinical results from trials addressing the impact of ICIs in AML have been globally disappointing (12). These data indicate the need for a more in-depth understanding of the complex cellular network operating within the AML immune microenvironment. Furthermore, considering that many studies have already shown that chemotherapy can be detrimental to the activity and survival of immune cells (13–16), a better characterization of the immunological effects of novel drugs is of utmost importance for fully exploiting immunotherapy strategies in AML.

The targeting of the anti-apoptotic B-cell lymphoma 2 (BCL-2) pathway has emerged as an efficacious and well-tolerated clinical strategy in AML. In particular, the remarkable results of Venetoclax (VEN), a specific BCL-2 inhibitor in combination with hypomethylating agents (HMAs) or low-dose cytarabine (17, 18), have led to the drug approval for the treatment of newly diagnosed unfit-for-chemotherapy AML patients (19). It is well-known that VEN causes leukemic cell apoptosis through several characterized mechanisms (20–23). However, VEN resistance can occur (24) and biomarkers able to predict response to treatment are still under investigation (25, 26). Furthermore, few studies analyzed the bystander effect of VEN on immune cells (27–29). In particular, it has been shown that VEN enhances T cell-mediated activity against AML cells both *in vitro* and *in vivo* through the generation of reactive oxygen species (ROS), and in parallel, azacitidine increases AML cell susceptibility to T cell-mediated cytotoxicity through viral mimicry (28). Furthermore, in AML patients, HMA and VEN treatment modulates the phenotype of NK and T cells, IFN-γ secretion by CD8^+^ T cells and Treg proliferation. Finally, an increased expression of perforin, CD39 and IFN-γ production by T cells as a pre-treatment signature is associated with VEN-resistance (29).

Despite its recognized importance for therapeutic purposes, the expression of several IC receptors on T cells has not been evaluated in AML patients after VEN treatment. In this study, we aimed to characterize the immunological repertoire of AML patients treated with VEN in combination with HMA by focusing on the modulation of IC receptors’ expression.

## Material and methods

### Patients and sample collection

The whole patient population included 27 patients, including 23 patients treated with the combination of HMAs and VEN and 4 patients with HMAs alone. The research was approved by the institutional review board of Area Vasta Emilia Centro (AVEC) Ethical Committee (approval code: 94/2016/O/Tess). Patients with AML were recruited at IRCCS Azienda Ospedaliero-Universitaria, Seràgnoli Hematology Institute in Bologna. Clinical samples and data were collected after written informed consent. According to normal clinical practice and to the study rationale, peripheral blood (PB) samples were collected from AML patients before the start of the treatment (baseline), and at the end of the first and second treatment cycle. Patients’ characteristics are summarized in **Table 1**. PB samples were used for flow cytometry analysis or centrifuged over a Ficoll-Hypaque gradient (Lympholyte CL5020, Cedarlane, Burlington, Canada) to collect mononuclear cells (MNCs), and then cryopreserved for sample collection and storage.

**Table 1:** Patient characteristics.

Patient ID	Sex	Age	Disease status	Therapy	Cytogenetics	Mutational status
AML 1	M	65	Relapse	DEC+VEN	Normal	Wild Type
AML 2	M	60	Relapse	DEC+VEN	Complex	*FLT3-ITD*
AML 3	F	70	Onset	AZA+VEN	Normal	Wild Type
AML 4	F	67	MRD	AZA+VEN	Normal	*NPM1; IDH1*
AML 5	M	67	Relapse	AZA+VEN	Inv(16)	*FLT3-TKD*
AML 6	M	60	Onset	DEC+VEN	Complex	*TP53*
AML 7	M	41	MRD	AZA+VEN	Normal	*NPM1*
AML 8	F	58	MRD	AZA+VEN	Normal	*NPM1; FLT3-TKD; IDH1*
AML 9	F	68	Onset	AZA+VEN	Normal	*NPM1*
AML 10	M	69	Onset	AZA+VEN	Normal	*IDH2*
AML 11	M	81	Onset	AZA+VEN	Trisomy (19)	Wild Type
AML 12	F	74	Onset	AZA+VEN	Monosomy (7)	Wild Type
AML 13	F	65	Onset (secondary to MDS)	AZA+VEN	Complex	*TP53*
AML 14	M	76	Onset	AZA+VEN	Complex	*TP53*
AML 15	M	79	Onset	AZA+VEN	Normal	*FLT3-ITD*
AML 16	M	71	Onset	AZA+VEN	Normal	Wild Type
AML 17	M	60	Onset	AZA+VEN	Complex	*IDH2*
AML 18	M	66	Onset	AZA+VEN	Normal	*NPM1*
AML 19	F	67	Onset	AZA+VEN	Normal	*NPM1; FLT3-ITD*
AML 20	F	69	Onset	AZA+VEN	Monosomy (7)	Wild Type
AML 21	F	80	Onset	AZA+VEN	Del (5q)	Wild Type
AML 22	M	70	Onset	DEC+VEN	Normal	NPM1; FLT3-ITD
AML 23	M	73	Onset	AZA+VEN	Normal	*FLT3-TKD*
AML 24	M	72	Onset	DEC	Normal	Wild Type
AML 25	M	86	Onset	AZA	Monosomy (7)	Wild Type
AML 26	M	84	Onset	AZA	Not Evaluable	Wild Type
AML 27	M	85	Onset	AZA	Normal	Wild Type

AML, acute myeloid leukemia; MRD, measurable residual disease; DEC, Decitabine; VEN, Venetoclax; AZA, Azacitidine.

### Flow cytometry analysis

Fresh PB was used for multi-parametric flow cytometry analysis. The monoclonal antibodies (mAbs) used for flow cytometry are listed in **Supplementary Table 1**. For the surface marker staining, PB samples containing at least 0.5x10^6^ white blood cells (WBCs) were labeled with dye-conjugated antibodies by incubation in the dark for 15 min at room temperature. Then, the red blood cells (RBC) were lysed, adding the FACS lysing solution (Beckton Dickinson Biosciences-BD, La Jolla, CA, USA), according to the manufacturer’s instruction.

The T cell compartment was monitored using the following markers: CD45, CD3, CD4, and CD8. The expression of CD45RA and CD197 (CCR7) was used to distinguish T-cell subsets defined as CD45RA^+^/CCR7^+^ (naïve=N); CD45RA^+^/CCR7^-^ (terminally differentiated=TD); CD45RA^-^/CCR7^+^ (central memory=CM); CD45RA^-^/CCR7^-^ (effector memory=EM) (**Supplementary Figure 1A**). Furthermore, we also evaluated the expression of the following exhaustion/senescent markers: CD279 (PD-1), TIM-3 (CD366), LAG-3, (CD223), CD244 (2B4), and CD57 and activating co-stimulatory ICs: OX40 (CD134) and ICOS (CD278). The Human Regulatory T Cell Whole Blood Staining kit (Thermo Fisher Scientific, Waltham, MA, USA) was used for the intracellular staining, according to the manufacturer’s instruction. Briefly, PB samples containing at least 1x10^6^ WBCs were stained for the surface markers in the dark for 15 min at room temperature. The RBC were then lysed for 20 minutes at room temperature using the RBC lysing solution. After 2 washes in PBS, the cells were resuspended in Fixation and Permeabilization Buffer for 30 minutes at 4°C. After 2 washes, cells were labeled with mAbs listed in **Supplementary Table 1** and incubated for 30 minutes at 4°C. Total Tregs were characterized as CD45^+^CD3^+^CD4^+^CD25^+/high^CD127^low/-^ cells. The following evaluation of CD25 and CD45RA expression dichotomizes FOXP3^+^ Tregs into 3 populations: CD25^+^/CD45RA^+^/FOXP3^+^ as naïve, CD25^high^/CD45RA^-^/FOXP3^high^ as effector and CD25^+^/CD45RA^-^/FOXP3^+^ as secreting non Tregs (**Supplementary Figure 1B**). Moreover, the expression of further markers, including ICOS, OX40, PD-1, and TIM-3, was assessed in the effector Treg population (30). Analysis was performed on a Cytoflex flow cytometer from Beckman Coulter, and results were obtained using the software analysis Kaluza 2.1 (Beckman Coulter, Brea, CA, USA).

### Statistical analysis

Statistical analyses were performed using GraphPad Prism software (v6.0). T-test and one-way ANOVA followed by Tukey’s multiple comparison *post hoc* test were used for comparison of groups. P value <0.05 was considered significant.

## Results

### Patients’ characteristics

To analyze VEN-dependent immune cell repertoire modifications, a group of 23 patients treated with the combination of VEN and HMAs (azacitidine or decitabine), hereafter defined as HMA plus VEN treatment and a control subgroup of 4 patients who received HMAs alone, were considered. Within the first group, 3 patients were enrolled and started therapy at relapse; 3 more patients received therapy in the phase of persistent MRD positivity, and 17 patients were included at disease onset. In the second group, the 4 patients were also enrolled at onset. Of note, regarding cytogenetics evaluation, 15 out of 27 patients had normal karyotype; 9 patients had high-risk cytogenetics; one was at low risk; one had trisomy 19, and one was not evaluable. Regarding molecular status, 7 patients were *NPM1* mutated, 4 had *FLT3-ITD* mutations, and 3 were *TP53* mutated (**Table 1**). The NGS analyses was used and performed using a 30 myeloid-related gene capture-based panel (Myeloid Solution, Sophia Genetics, Switzerland) on a Miseq instrument (Illumina, San Diego, California). The following genes and exons are included in the panel: *ABL1* (4-9), *ASXL1* (9,11,12,14), *BRAF* (15), *CALR* (9), *CBL* (8,9), *CEBPA* (all), *CSF3R* (all), *DNMT3A* (all), *ETV6* (all), *EZH2* (all), *FLT3* (13-15,20), *HRAS* (2,3), *IDH1* (4), *IDH2* (4), *JAK2* (all), *KIT* (2,8-11,13,17,18), *KRAS* (2,3), *MPL* (10), *NPM1* (10,11), *NRAS* (2,3), *PTPN11* (3,7-13), *RUNX1* (all), *SETBP1* (4), *SF3B1* (10-16), *SRSF2* (1), *TET2* (all), *TP53* (2-11), *U2AF1* (2,6), *WT1* (6-10), *ZRSR2* (all). The platform Sophia DDM (Sophia Genetics) was used for bio-informatic data analysis. Intronic and synonymous variants were filtered out and variants present with a minor allele frequency (MAF) >1%, according to population databases (ExAc, 1000 genomes), were considered polymorphic changes. Variants were also filtered according to the variant allele frequency (VAF): all variants with VAF ≥5% were reported. COSMIC and VARSOME databases, as well as in silico functional predictors (SIFT, PolyPhen) were used for variant interpretation; only variants described as pathogenic or potentially pathogenic were reported.

Eighteen patients received azacitidine, while 5 patients were treated with decitabine. Furthermore, 7 patients from the first group, treated with HMA plus VEN, were consolidated with allogeneic hematopoietic stem cell transplant (HSCT).

### HMA plus VEN modifies T cell subsets

**Figure 1:**
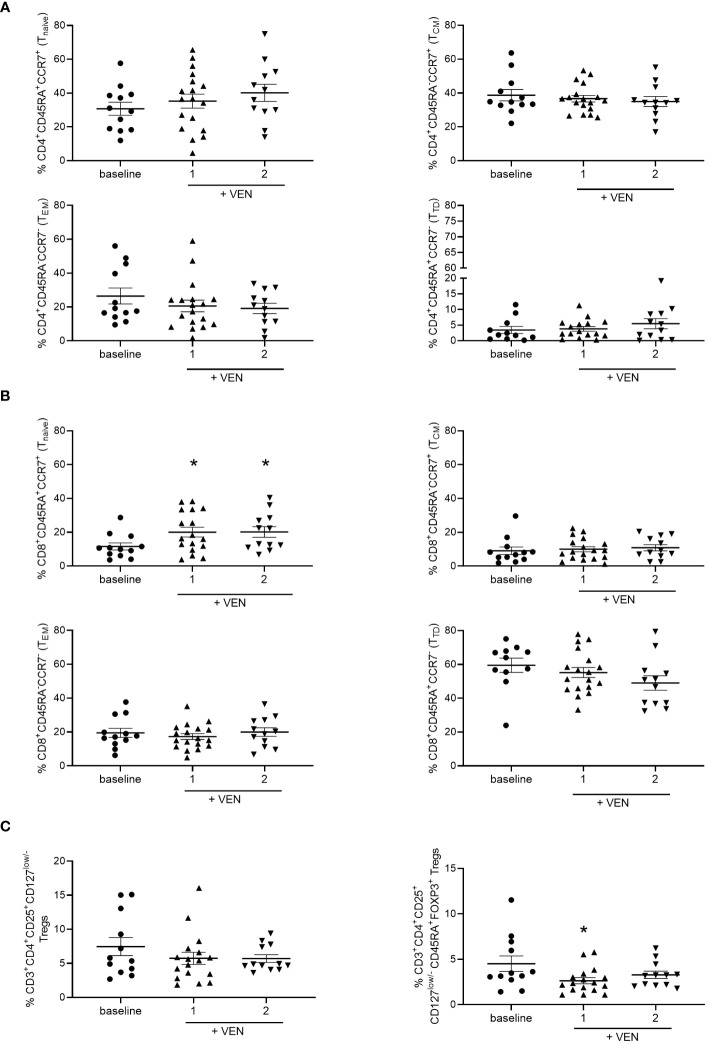
Distribution of T cell subsets during HMA plus VEN treatment. All the samples were analyzed by flow cytometry and data are represented as mean ± SEM. **(A)** CD4^+^ T cell subsets. Frequencies of CD45RA^+^/CCR7^+^ cells (naïve=N, upper left panel), CD45RA^-^/CCR7^+^ cells (central memory=CM, upper right panel); CD45RA^-^/CCR7^-^ cells (effector memory=EM, lower left panel), CD45RA^+^/CCR7^-^ cells (terminally differentiated=TD, lower right panel). Baseline, at least n=11; cycle 1 at least, n=16; cycle 2, n=12. **(B)** CD8^+^ T cell subsets. Cells were analyzed as described for A). Baseline, at least n=11; cycle 1, at least n=17; cycle 2, n=12. For naïve CD8^+^ T cells (upper left panel): baseline vs cycle 1, *P=0.037; baseline vs cycle 2, *P=0.034. **(C)** Frequencies of Tregs (CD3^+^CD4^+^CD25^+^CD127^low/-^ cells) before treatment (n=12), after first (n=17), and second (n=12) cycle of HMA plus VEN (left panel). Frequencies of cytokine secreting non-suppressive Tregs (CD45RA^-^CD25^high^FOXP3^+^ cells; right panel) within the CD3^+^CD4^+^CD25^+^CD127^low/-^ Treg population, expressed as a percentage of CD4^+^ cells, in patients before treatment (baseline, n=12), after first (n=17), and second (n=12) cycle of HMA plus VEN; baseline vs cycle 1 *P=0.041.

To evaluate the effects of the treatment of HMA plus VEN on circulating immune cell repertoire, we first analyzed CD4^+^ and CD8^+^ T cell subsets in the PB of AML patients during treatment, compared to PB examined before treatment (baseline). Total T lymphocytes were slightly increased, although not significantly, after the first and second cycles of HMA plus VEN treatment, compared to baseline (**Supplementary Figure 2A**). Focusing on T cell subsets, we found that the percentage of CD4^+^ and CD8^+^ T cells did not change during HMA plus VEN treatment (**Supplementary Figures 2B, C**). A more detailed analysis of effector CD4^+^ T-cell subpopulations, identified as naïve/central memory/effector memory/terminally differentiated by CD45RA and CCR7 expression, did not show significant changes during HMA plus VEN treatment, compared to baseline (**Figure 1A**). Interestingly, regarding CD8^+^ T-cell subpopulations, we found a significant increase of CD8^+^ naïve T cells during the first and second cycles of HMA plus VEN treatment (mean ± SEM, baseline: 11.56 ± 2.07; cycle 1: 20.04 ± 2.89; cycle 2: 20.14 ± 3.18; baseline vs cycle 1, *P=0.037; baseline vs cycle 2, *P=0.034), while the memory subsets were not modified (**Figure 1B** left panel). Finally, we also studied the Treg population, and found that total Tregs, identified as CD4^+^CD25^+^CD127^low/-^ T cells, tended to decrease after HMA plus VEN treatment (**Figure 1C** left panel). A more detailed analysis of Treg subsets revealed that the HMA plus VEN only slightly affected the naive and effector Treg distribution (**Supplementary Figures 2D, E**). On the contrary, the percentage of cytokine-secreting non-suppressive Tregs, which includes Th17 cells, was significantly decreased after the first cycle of HMA plus VEN treatment, compared to the baseline (mean ± SEM, baseline: 4.51% ± 0.855%; cycle 1: 2.63% ± 0.34%; cycle 2: 3.28% ± 0.40%; baseline vs cycle 1, *P=0.041; **Figure 1C** right panel).

These data suggest that HMA plus VEN treatment induces a redistribution of immune cell subsets towards a more naïve phenotype for CD8^+^ T-cell subsets. On the contrary, HMA plus VEN treatment does not significantly modify the Treg compartment, while it induces a decrease in the percentage of cytokine-secreting non-suppressive Tregs.

### HMA plus VEN treatment alters the expression of PD-1 and TIM-3 on CD4^+^ and CD8^+^ T cells

**Figure 2:**
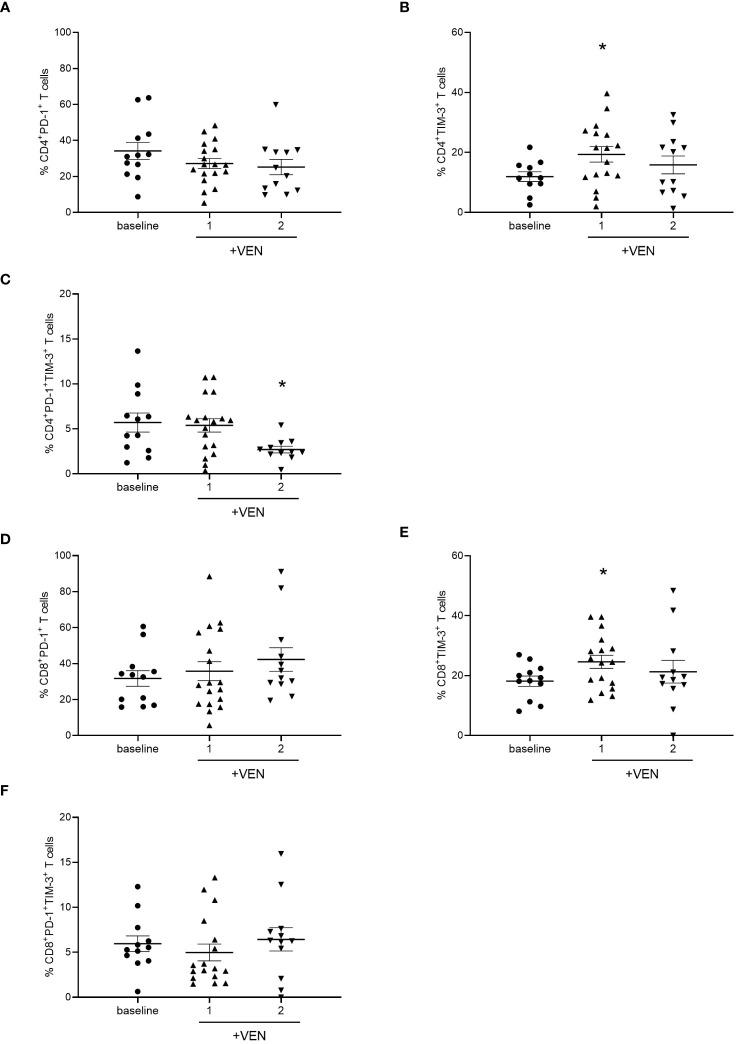
Analysis of ICs on T cells during HMA plus VEN treatment. All the samples were analyzed by flow cytometry and data are represented as mean ± SEM. **(A)** Percentage of CD4^+^PD-1^+^ T cells before treatment (n=12), after first (n=18), and second (n=12) cycle of HMA plus VEN. **(B)** Percentage of CD4^+^ TIM-3^+^ T cells before treatment (n=12), after first (n=17) and second (n=12) cycle of HMA plus VEN (baseline vs cycle 1, *P=0.041. **(C)** Percentage of PD-1^+/^TIM-3^+^ CD4^+^ T cells before treatment (n=12), after first (n=18), and second (n=11) cycle of HMA plus VEN, baseline vs cycle 1, *P=0.017. **(D)** Percentage of CD8^+^PD-1^+^ T cells before treatment (n=12), after first (n=18), and second (n=12) cycle of HMA plus VEN. **(E)** Percentage of CD8^+^ TIM-3^+^ T cells before treatment (n=12), after first (n=17), and second (n=12) cycle of HMA plus VEN. Baseline vs cycle 1, *P=0.039. **(F)** Percentage of PD-1^+/^TIM-3^+^ CD8^+^ T cells before treatment (n=12), after first (n=17), and second (n=12) cycle of HMA plus VEN.

Then, we evaluated the effects of HMA plus VEN treatment on the expression of IC inhibitory receptors, such as PD-1, TIM-3, and LAG-3, and activating co-stimulatory molecules, including ICOS and OX-40. Interestingly, we found that HMA plus VEN treatment induced changes in the percentage of PD-1^+^ and TIM-3^+^ CD4^+^ T cells compared to baseline (**Figure 2**). In particular, the percentage of PD-1^+^ CD4^+^ T cells was slightly reduced after the first cycle of HMA plus VEN treatment, while TIM-3^+^ CD4^+^ T cells were significantly increased after the first cycle (mean ± SEM, baseline: 11.32% ± 1.63%; cycle 1: 19.33% ± 2.55%; cycle 2: 15.86% ± 2.97%; Baseline vs cycle 1, *P=0.041), as shown in **Figures 2A, B**, respectively. PD-1^+^/TIM-3^+^ T cells showed a statistically significant decrease after the second cycle of HMA plus VEN treatment, compared to baseline (mean ± SEM, baseline: 5.07% ± 1.06%; cycle 1: 5.39% ± 0.74%; cycle 2: 2.70% ± 0.37%; baseline vs cycle 1, *P=0.017; **Figure 2C**). Of note, VEN plus HMA treatment did not change the expression of LAG-3, OX40 and ICOS on CD4^+^ T cells (data not shown).

We next analyzed the expression of IC receptors on CD8^+^ T cells, and we found a slight not significant increase in PD-1 expression after the second cycle of HMA plus VEN treatment (**Figure 2D**). Similar to what we found for CD4^+^ T cells, TIM-3^+^ CD8^+^ T cells resulted significantly increased after the first cycle of HMA plus VEN treatment, compared to baseline (mean ± SEM, baseline: 18.17% ± 1,70%; cycle 1: 24.60% ± 2.18%; cycle 2: 21.31% ± 3.78%; baseline vs cycle 1, *P=0.039; **Figure 2E**). At the same time, we found a trend toward a decrease in the PD-1^+^/TIM-3^+^ CD8^+^ T cells after the first treatment cycle (**Figure 2F**).

**Figure 3:**
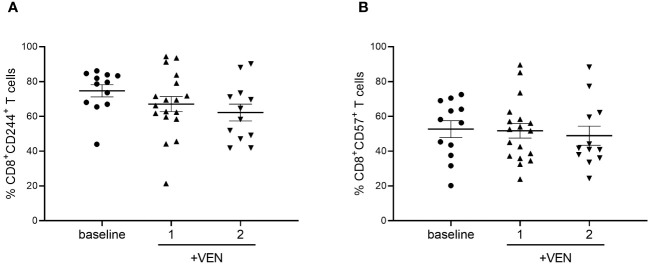
Expression of CD244 and CD57 on CD8^+^ T cells during HMA plus VEN treatment. All the samples were analyzed by flow cytometry and data are represented as mean ± SEM. **(A)** Percentage of CD8^+^CD244^+^ T cells before treatment (n=12), after first (n=18), and second (n=12) cycle of HMA plus VEN. **(B)** Percentage of CD8^+^ CD57^+^ T cells before treatment (n=12), after first (n=18), and second (n=12) cycle of HMA plus VEN.

To further characterize the expression of markers involved in T cells’ exhausted and senescent status in AML patients treated with HMA plus VEN, we analyzed the expression of CD244 and CD57 on CD4^+^ and CD8^+^ T cells. No significant differences were found after HMA plus VEN treatment (**Figures 3A, B**). Regarding Tregs, the expression of CTLA-4, OX-40, ICOS, PD-1 and TIM-3 was analyzed in the effector Tregs, known to have the highest suppressive potential among Treg subsets (30), but we did not find any differences in their expression after treatment (**Supplementary Figure 3**).

Overall, these findings indicate that HMA plus VEN modify the expression of ICs on CD4^+^/CD8^+^ T cells but not in Treg cells.

### VEN specifically decreases cytokine-secreting non-suppressive Tregs and regulates TIM-3 expression on CD8^+^ and PD-1/TIM-3 expression on CD4^+^ and CD8^+^ T cells

**Figure 4:**
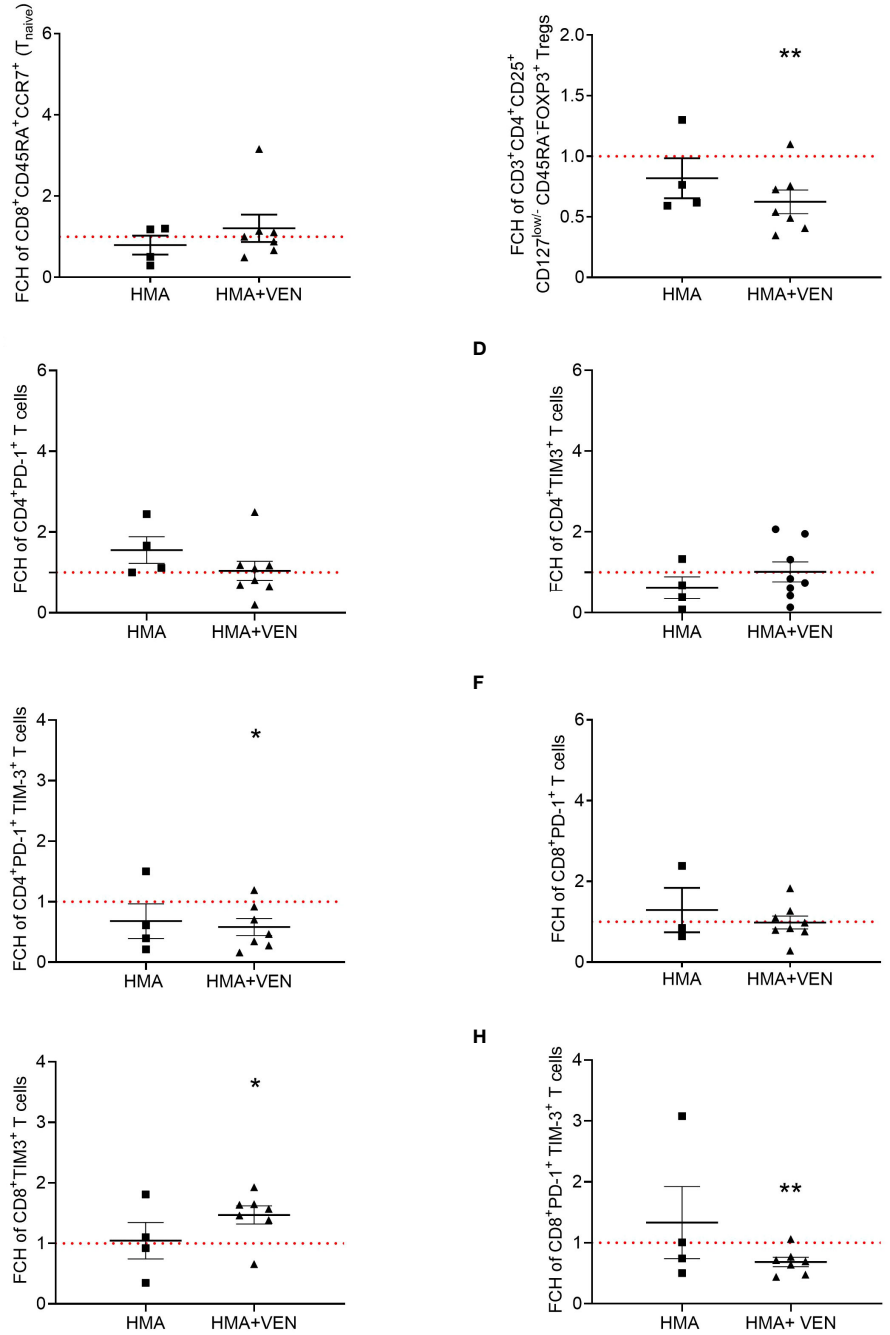
Analysis of pair-matched patients. All the samples were collected from patients during only 1 cycle of HMA (grey) or 1 cycle of HMA plus VEN treatment (black), and were analyzed by flow cytometry. Fold change percentages (FCH) were calculated normalizing the baseline value to 1 shown as the red line (cycle 1/baseline), and data are represented as mean ± SEM. **(A)** FCH of CD45RA^+^/CCR7^+^ cells (naïve) CD8^+^ T cells before and after first treatment cycle (HMA, n=4; HMA plus VEN, n=7). **(B)** FCH of cytokine secreting non-suppressive Tregs (CD45RA^-^FOXP3^+^ cells) within the CD3^+^CD4^+^CD25^+^CD127^low/-^ Treg population, expressed as a percentage of total CD4^+^ T cells (HMA, n=4; HMA plus VEN, n=7; baseline vs cycle 1 HMA plus VEN, **P=0.008). **(C)** FCH of CD4^+^PD-1^+^ T cells before and after first treatment cycle (HMA, n=4; HMA plus VEN, n=8). **(D)** FCH of CD4^+^ TIM-3^+^ T cells before and after first treatment cycle (HMA, n=4; HMA plus VEN, n=8). **(E)** FCH of CD4^+^ PD-1^+^TIM-3^+^ T cells before and after first treatment cycle (HMA, n=4; HMA plus VEN, n=7; baseline vs cycle 1 HMA plus VEN, *P=0.025). **(F)** FCH of CD8^+^PD-1^+^ T cells before and after first treatment cycle (HMA, n=3; HMA plus VEN, n=8). **(G)** FCH of CD8^+^TIM-3^+^ T cells before and after first treatment cycle (HMA, n=4; HMA plus VEN, n=7; baseline vs cycle 1 HMA plus VEN, *P=0.020). **(H)** FCH of CD8^+^PD-1^+^TIM-3^+^ T cells before and after first treatment cycle (HMA, n=4; HMA plus VEN, n=7; baseline vs cycle 1 HMA plus VEN, **P=0.007).

To dissect the specific contribution of VEN in modulating the immune cell repertoire of AML patients treated with HMA plus VEN, we considered pair-matched samples comparing the time points before treatment (baseline considered as 1), treated only with HMA and treated with the combination of HMA plus VEN. Focusing on T lymphocyte subsets, we found that VEN did not change the distribution of CD8^+^ naïve T cells (**Figure 4A**). In contrast, VEN specifically induced a significant reduction in the percentage of cytokine-secreting non-suppressive Tregs (mean ± SEM, HMA+VEN cycle 1: 0.62% ± 0.1%; baseline vs cycle 1 HMA plus VEN, **P=0.008; **Figure 4B**).

Next, we analyzed the IC expression and, interestingly, in contrast to what was observed considering the entire group of patients, we found that the expression of PD-1 on CD4^+^ T cells was slightly decreased by VEN treatment for matched patients (**Figure 4C**). A similar pattern was observed for TIM-3 expression on CD4^+^ T cells, whose expression was not affected by VEN and seemed to decrease after HMA treatment (**Figure 4D**). On the contrary, we observed a significant decrease of CD4^+^ T cells expressing TIM-3 and PD-1 only in patients treated with the combination HMA plus VEN after one cycle of HMA (mean ± SEM, HMA+VEN cycle 1: 0.58% ± 0.14%; baseline vs cycle 1 HMA plus VEN, *P=0.025; **Figure 4E**). Next, we analyzed the expression of ICs on CD8^+^ T cells, and we found that PD-1 expression is not influenced by VEN (**Figure 4F**). Conversely, the expression of TIM-3 remained stable after HMA treatment only, but was significantly increased after HMA plus VEN treatment indicating a VEN-specific effect (mean ± SEM, HMA+VEN cycle 1: 1.47 ± 0.15%; *P=0.020; **Figure 4G**). Interestingly, VEN treatment specifically and significantly decreased the expression of PD-1/TIM-3 on CD8^+^ T cells (**Figure 4H**; mean ± SEM, HMA+VEN cycle 1: 0.68 ± 0.08%; baseline vs cycle 1 HMA plus VEN, **P=0.007). Importantly, we found a statistically significant difference between the percentage of CD8^+^Tim-3^+^ T cells at baseline and therapy response after the first cycle (**Supplementary Figure 4**). In particular, the increased percentage of Tim-3^+^CD8^+^ T cells was positively correlated with complete remission (CR) compared to the patients which did not achieve CR (**Supplementary Figure 4A**). The population of CD8^+^ T cells co-expressing PD-1 and Tim-3, strongly associated with exhaustion status, was not correlated with therapy response (**Supplementary Figure 4B**).

Overall, an in-depth analysis performed comparing samples collected after HMA only and at the first cycle of HMA plus VEN treatment with respect to the baseline, suggested a specific role of VEN in modulating the expression of inhibitory IC receptors on both CD4^+^ and CD8^+^ T cells. In particular, VEN induces an increase of the TIM-3^+^ CD8^+^ T cell population and a reduction of PD-1^+/^TIM-3^+^ CD4^+^ and CD8^+^ T cells.

## Discussion

In this study, we analyzed the immune cell repertoire of 23 AML patients treated with HMA plus VEN. We focused on the modulation by treatment of IC receptors’ expression on T cells. Our data unravel the ability of VEN to modulate the immune cell compartment and the expression of ICs.

While the HMA plus VEN treatment is becoming the backbone for AML therapy, new combinatorial drug strategies, including immunotherapies, have been considered to improve the survival of AML patients. For this reason, increased knowledge of the immunological effects of VEN treatment is fundamental for fully exploiting new immunotherapy strategies in AML.

VEN is a specific BCL-2 inhibitor. Interestingly, various T cell subsets depend on BCL-2 for their survival to varying degrees and, thus, may be influenced by the treatment with VEN (31). In particular, naïve and memory T cells require BCL-2 for survival and/or homeostasis (32, 33). In our study, after HMA plus VEN treatment, we did not find a modulation of either the total percentage of CD3^+^, CD4^+^ and CD8^+^ T cells nor the percentage of CD4/CD8 immune T cell subsets, except for an increase of naïve CD8^+^ T cells. This finding contrasts with previous works showing that BCL-2 inhibition in different settings led to a reduction of total and naïve T cells and to an increase in the proportion of memory T cells (34–36). In particular, in AML patients treated with HMA and VEN a depletion of total T, B and NK cells and an increase of the CD4^+^ and CD8^+^ T-cell frequencies with an effector memory phenotype at the expense of naïve T cells have been observed (29). However, despite a decrease in the total number of CD4^+^ and CD8^+^ T cells, their differentiation status did not change after 1 year of VEN-based therapy in patients with chronic lymphocytic leukemia (27). The discrepancy in these findings could be due to different reasons:1) the different timing of blood collection during the therapy; 2) the analysis of percentages compared to the absolute numbers, and 3) the different cell surface markers used to identify the T cell immune subsets. Furthermore, it must be considered that our particular and previously unexplored setting, i.e. the paired analysis conducted on patients treated with HMA plus VEN or HMA only, may have allowed us to distinguish a VEN-dependent effect from an HMA-dependent effect. In our hands, VEN did not alter the percentage of CD8^+^ naïve T cells.

Next, we found that HMA plus VEN treatment did not affect total Tregs and their subsets, according with a previous study (29). These results are corroborated by the demonstration that Tregs are relatively resistant to apoptosis induced by BCL-2 inhibition compared to other T cells. Indeed, BCL-2 is not required for Treg survival, as opposed to MCL-1, another member of the BCL-2 pathway, the loss of which leads to fatal autoimmunity in a mouse model (37). Interestingly, by analysing Tregs’ subset after HMA plus VEN treatment, we found a decrease in the CD25^+^/CD45RA^-^/FOXP3^+^ T cells, which represent a cytokine-secreting and non-suppressive population with Th17 cell potential (30). Of note, we did not observe such reduction in patients treated with HMA only, suggesting a putative-specific role of VEN.

To our knowledge, this is the first study reporting the effect of VEN treatment on this cell population. The plasticity of CD4^+^ T cell subsets is well-known, and in particular, the plasticity between Th17 and Tregs has been described (38). The Th17 and Treg differentiation networks play a critical role in the development of autoimmune diseases (39). Interestingly, Th17 cells are considered a double-edged sword in solid tumors, promoting at the same time tumor progression, angiogenesis, and anti-tumor response, and immune cells’ recruiting (40, 41). In line with this, Th17 cells can promote both pro- and anti-tumor immunity in hematological malignancies. An increase in Th17 cells was associated both with better and worse clinical outcomes in AML patients (42–44). Moreover, an increase in Th17 cells population was found in not responding relapsed/refractory AML patients, compared to responders treated with HMA and the anti-PD-1 Nivolumab (45).

By specifically addressing the expression of inhibitory and stimulatory IC receptors, we found a significant increase in TIM-3^+^CD4^+^ T cells after the first cycle of treatment. However, when we paired patients to dissect the contribution of VEN, this finding was not confirmed. On the contrary, we found a VEN-specific increased expression of TIM-3 on CD8^+^ T cells after the first cycle. As previously mentioned, TIM-3 expression has been associated with exhaustion and dysfunction of T cells, and with immune evasion in AML (46). Interestingly, AML cells can enhance the expression of inhibitory receptors, including TIM-3, thus determining CD4^+^ T cell exhaustion *in vitro* (47), and TIM-3 expression on immune cells has been correlated to both a better and a poor prognosis in AML patients (48–50). Besides its expression on immune cells, TIM-3 has been found in AML leukemia stem cells but not in their normal counterparts (51, 52), and increased levels of Galectin- 9, a TIM-3 ligand, were found in AML patients’ serum (53). However, despite its implications in cell survival and disease progression, the role of TIM-3 on AML cells is still unknown and the blockade of TIM-3 as a single agent did not show a substantial clinical benefit. However, our finding showing the increased TIM-3 expression on CD8^+^ T cells after VEN treatment could support on-going clinical trials based on the use of TIM-3 inhibitor (NCT03066648, NCT04150029, NCT04623216) in combination with VEN.

Of note, we found a significant VEN-dependent decrease of PD-1^+^TIM-3^+^ CD4^+^ and CD8^+^ T cells after the first treatment cycle. PD-1^+^TIM-3^+ ^ double-positive T cells have been indicated as prognostic in different settings. The exhausted T cell population PD-1^high^TIM-3^+^ was shown to be functionally deficient and was associated with AML relapse after allogeneic HSCT (54). A higher proportion of PD-1^+^TIM-3^+ ^ CD3^+ ^ T cells in the bone marrow and of PD-1^+ ^ TIM-3^+ ^ CD4^+ ^ T cells in the PB of non-complete remission versus CR patients after first-cycle chemotherapy were observed (55). In mouse model, the PD-1 and TIM-3 co-expression on CD8^+^ T cells was increased during AML progression (56). Within the CD8^+^ PD-1^+^ T cells, the TIM-3 expression identifies the most dysfunctional T-cell population in human chronic viral infections (57, 58). This finding could indicate a beneficial effect of VEN on functional T-cell response confirming a previous study showing that VEN, leading to increased ROS generation, enhances T cell-mediated cytotoxicity against AML cells *in vitro* and *in vivo* (28).

In conclusion, our study had new evidence in support of the ability of VEN to modulate immune cell composition and phenotype. In particular, we identified two significant VEN-specific effects 1) a decrease of cytokine-secreting non-suppressive Tregs with Th17 cell potential, the clinical significance of which deserves to be explored; 2) a decrease of the PD-1^+^/TIM-3^+^ CD4^+^ and CD8^+^ T cells, which may have a beneficial effect. Furthermore, we also found an enhanced expression of TIM-3 on CD8^+^ T cells. Understanding the immunological microenvironment under the action of the selective BCL-2 inhibition can potentially reveal both novel mechanisms of resistance and new treatment combinations.

## Data availability statement

The original contributions presented in the study are included in the article/**Supplementary Material**. Further inquiries can be directed to the corresponding author.

## Ethics statement

The studies involving humans were approved by The institutional review board of Area Vasta Emilia Centro (AVEC) Ethical Committee (approval code: 94/2016/O/Tess). The studies were conducted in accordance with the local legislation and institutional requirements. The participants provided their written informed consent to participate in this study.

## Author contributions

GC: Data curation, Formal analysis, Methodology, Writing – original draft, Writing – review & editing. DF: Writing – review & editing, Formal analysis. GC: Resources, Writing – review & editing. AP: Formal analysis, Writing – review & editing. MC: Writing – review & editing. VS: Formal analysis, Writing – review & editing. LB: Methodology, Writing – review & editing. VR: Methodology, Writing – review & editing. EO: Methodology, Writing – review & editing. MC: Funding acquisition, Writing – review & editing. DO: Conceptualization, Supervision, Writing – review & editing. AC: Conceptualization, Supervision, Writing – review & editing.
