# Supplementary material for: Ex vivo characterization of acute myeloid leukemia patients undergoing hypomethylating agents and venetoclax regimen reveals a venetoclax-specific effect on non-suppressive regulatory T cells and bona fide PD-1+TIM3+ exhausted CD8+ T cells
Source: Front Immunol. 2024 May 15;15:1386517. doi: 10.3389/fimmu.2024.1386517 (PMC11133521; doi:10.3389/fimmu.2024.1386517)
Supplement: Supplementary file 1 [file DataSheet_1.doc]

# Supplementary Material

**EX VIVO CHARACTERIZATION OF ACUTE MYELOID LEUKEMIA PATIENTS UNDERGOING HYPOMETHYLATING AGENTS AND VENETOCLAX REGIMEN REVEALS A VENETOCLAX-SPECIFIC EFFECT ON NON-SUPPRESSIVE REGULATORY T CELLS AND *BONA FIDE* PD-1^+^TIM3^+^ EXHAUSTED CD8^+^ T CELLS**

**Corradi et al.**

**Supplementary Figures and Tables**

**Supplementary Figure 1.**


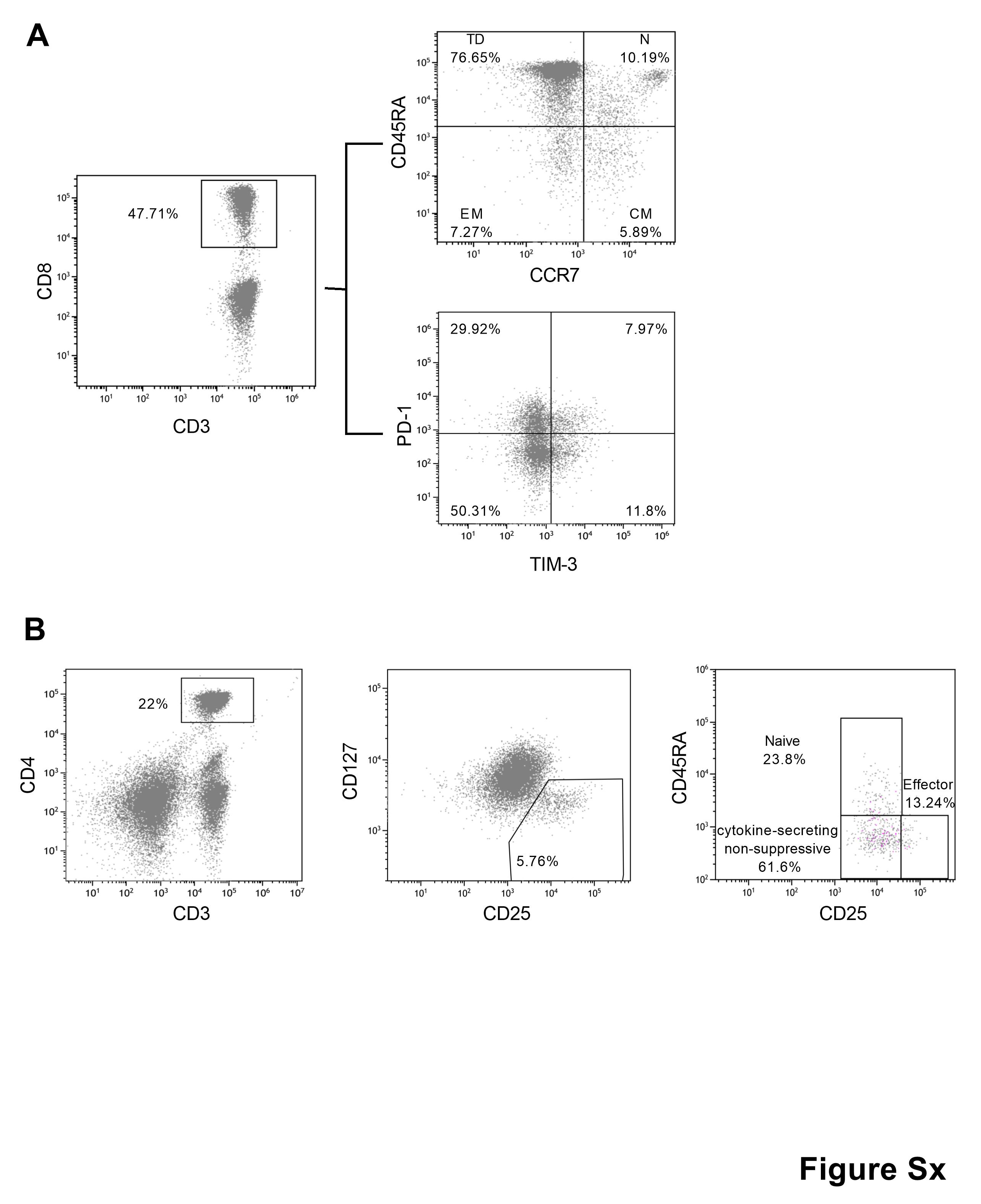


**Supplementary Figure 1. The gating strategy of T effector and regulatory cells.** Flow cytometry gating strategy of one representative sample. **A)** CD8 effector T cells and their subpopulations were identified as CD45RA^+^/CCR7^+^ (naïve=N), CD45RA^-^/CCR7^+^ (central memory=CM), CD45RA^-^/CCR7^-^ (effector memory=EM), and CD45RA^+^/CCR7^-^ (terminally differentiated=TD) cells-upper panel. The expression of PD-1 and Tim-3 on CD8 effector T cells-lower panel. **B)** Total Tregs **(**CD3^+^CD4^+^CD25^+^CD127^low/-^) and their subpopulations identified as naïve Tregs (CD45RA^+^CD25^+^FOXP3^+^ cells), effector Tregs (CD45RA^-^CD25^high^FOXP3^+^ cells), and cytokine-secreting non-suppressive Tregs (CD45RA^-^CD25^+^FOXP3^+^ cells).

**Supplementary Figure 2.**

**
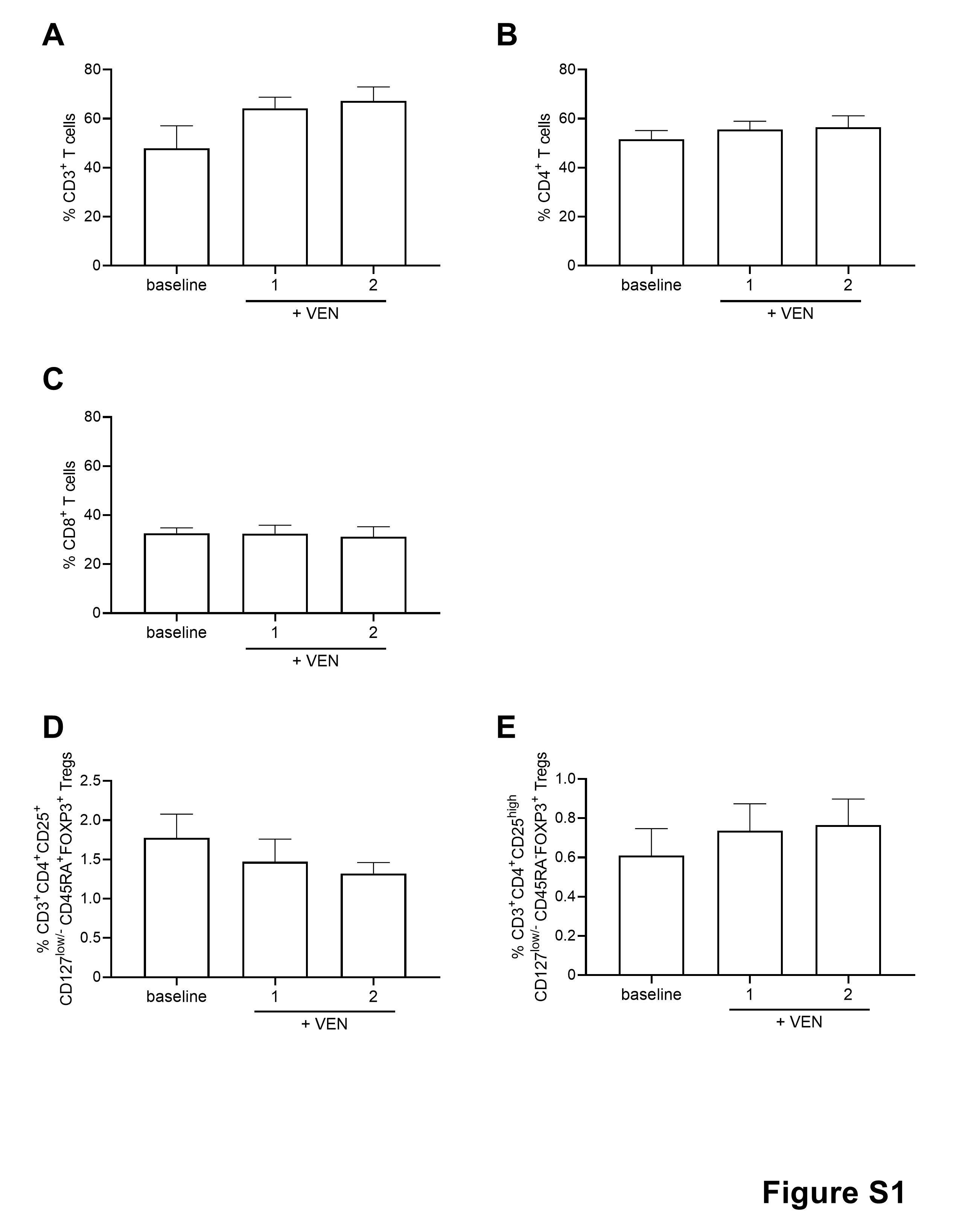
**

**Supplementary Figure 2. Distribution of T lymphocytes and Treg subsets during HMA plus VEN treatment.** All the samples were analyzed by flow cytometry and data are represented as mean ± SEM. **A)** Percentage of CD45^+^CD3^+^ T cells, gated on singlets, before treatment (n=12), after first (n=18), and second (n=12) cycle of HMA plus VEN. **B)** Frequency of CD4^+^ T cells, gated on CD3^+^, before treatment (n=12), after first (n=18), and second (n=12) cycle of HMA plus VEN. **C)** Percentage of CD8^+^ T cells, gated on CD3^+^, before treatment (n=12), after first (n=18), and second (n=12) cycle of HMA plus VEN. **D)** Frequencies of naïve Tregs (CD45RA^+^FOXP3^+^ cells) within the CD3^+^CD4^+^CD25^+^CD127^low/-^ Treg population, expressed as a percentage of total CD4^+^ T cells, in patients before treatment (n=12), after first (n=15), and second (n=11) cycle of HMA plus VEN. **E)** Percentage of effector Tregs (CD45RA^-^CD25^high^FOXP3^+^ cells) within the CD3^+^CD4^+^CD25^+^CD127^low/-^ Treg population, expressed as a percentage of total CD4^+^ T cells, in patients before treatment (n=11), after first (n=16), and second (n=12) cycle of HMA plus VEN.

**Supplementary Figure 3.**

**
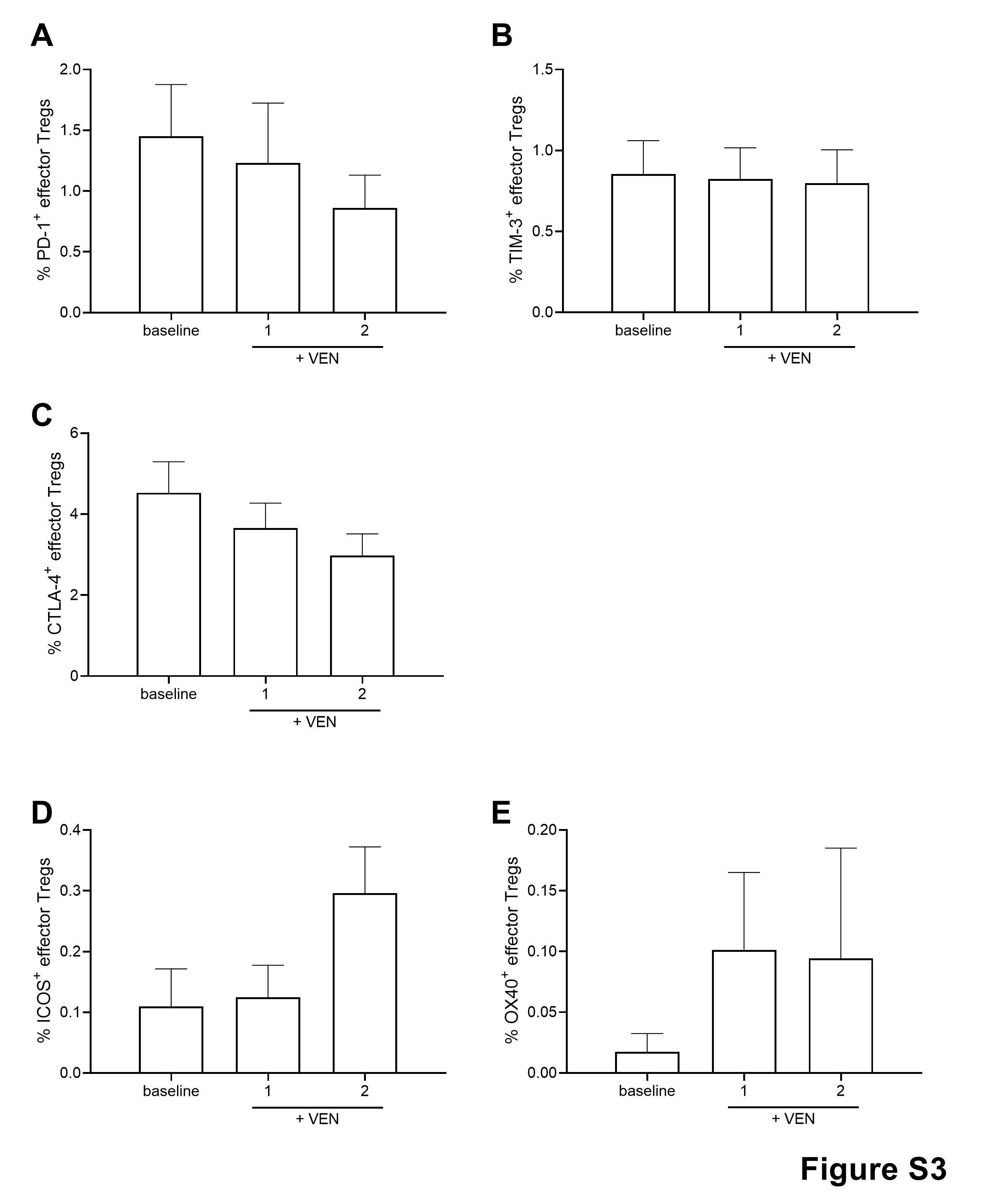
**

**Supplementary Figure 3. Analysis of ICs on effector Tregs during HMA plus VEN treatment.** All the samples were analyzed by flow cytometry and data are represented as mean ± SEM. **A)** Percentage of PD-1^+^ effector Tregs (CD45RA^-^CD25^high^FOXP3^+^ cells) within the CD3^+^CD4^+^CD25^+^CD127^low/-^ Treg population, expressed as a percentage of total CD4^+^ T cells, in patients before treatment (n=12), after first (n=16), and second (n=12) cycle of HMA plus VEN. **B)** Frequency of TIM-3^+^ effector Tregs, identified as described above, in patients before treatment (n=11), after first (n=15), and second (n=12) cycle of HMA plus VEN. **C)** Percentage of CTLA-4^+^ effector Tregs, identified as described above, in patients before treatment (n=12), after first (n=17), and second (n=12) cycle of HMA plus VEN. **D)** Frequency of ICOS^+^ effector Tregs in patients before treatment (n=10), after first (n=15), and second (n=11) cycle of HMA plus VEN. **E)** Percentage of OX40^+^ effector Tregs in patients before treatment (n=12), after first (n=15), and second (n=9) cycle of HMA plus VEN.

**Supplementary Figure 4.**


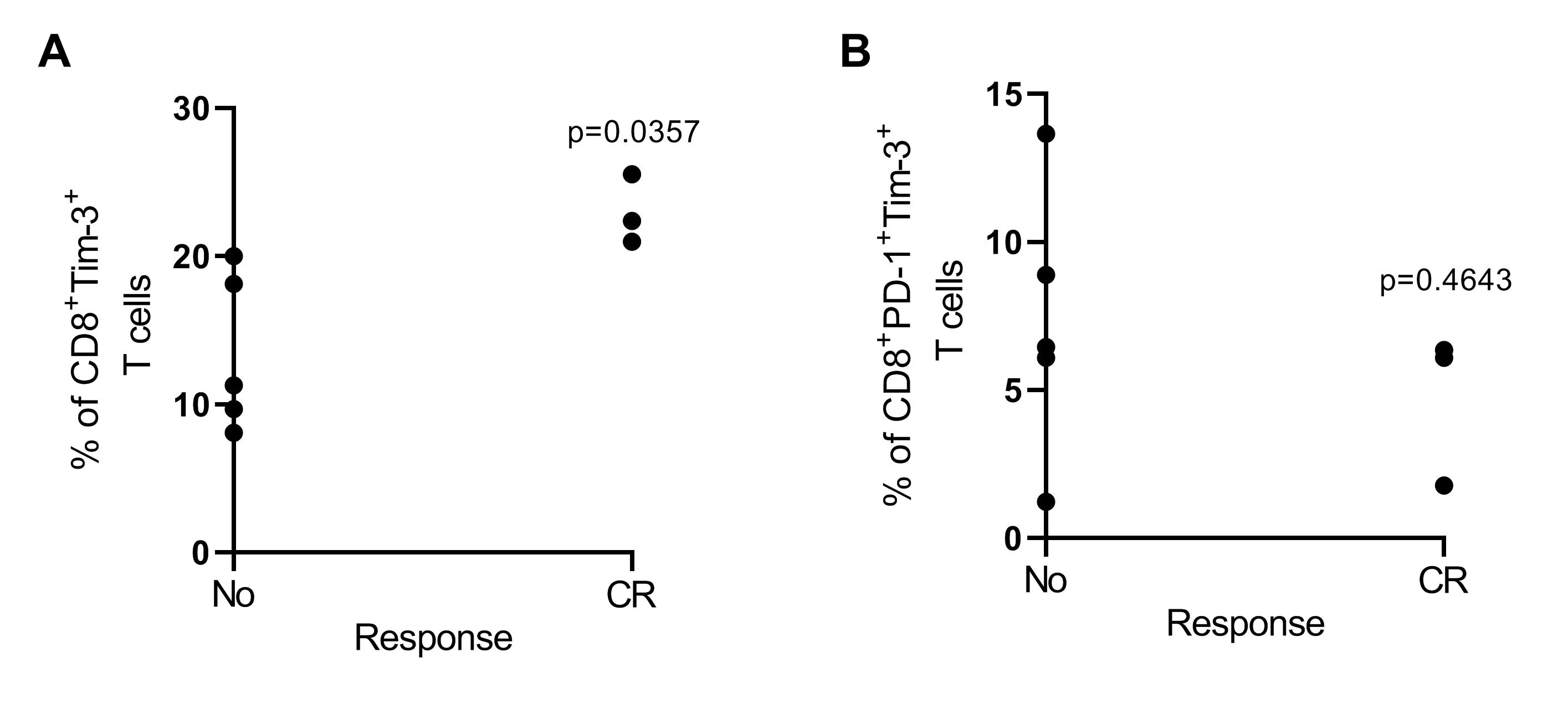


**Supplementary Figure 4. Correlation analysis of Tim-3 and PD-1 expression on CD8^+^ T cells with response to VEN treatment.** The frequencies of Tim-3 (**A**) or PD-1/Tim-3 (**B**) expression on CD8^+^ T cells at baseline were analyzed by Spearman correlation analysis with VEN treatment response after first cycle. CR=complete remission; No=stable disease or partial response; n=5

**Supplementary Table 1.** Primary antibodies used in this study for flow cytometry.

| **Antigen (host species)** | **Species reactivity** | **Clone or class** | **Conjugate** | **Dilution** | **Source** |
| --- | --- | --- | --- | --- | --- |
| CD127 (mouse) | human | A019D3 | PerCP-Cy5.5 | 1:100 | BioLegend |
| CD25 (mouse) | human | BC96 | PE-Cy7 | 1:100 | BioLegend |
| CD3 (mouse) | human | SK7 | APC-H7 | 1:100 | BD Pharmingen |
| CD4 (mouse) | human | SK3 | Pe-Cy7 | 1:100 | Invitrogen |
| CD4 (mouse) | human | SK3 | APC | 1:100 | BD |
| CD45 (mouse) | human | 2D1 | Alexa Fluor700 | 1:100 | BioLegend |
| CD45RA (mouse) | human | HI100 | BV510 | 1:100 | BioLegend |
| CD8 (mouse) | human | HIT8a | PE | 1:100 | eBiosciences |
| CD8 (mouse) | human | SK1 | APC | 1:25 | eBiosciences |
| CTLA-4 (mouse) | human | BNI3 | PE | 1:100 | BD Pharmingen |
| FOXP3 (mouse) | human | 236A/E7 | V450 | 1:100 | BioLegend |
| PD-1 (mouse) | human | EH12.2H7 | BV421 | 1:100 | BioLegend |
| PD-1 (mouse) | human | EH12 | BB515 | 1:100 | BD Horizon |
| TIM-3 (mouse) | human | 7D3 | PE | 1:100 | BD Pharmingen |
| LAG-3 (mouse) | human | 11C3C65 | FITC | 1:100 | BioLegend |
| OX40 (mouse) | human | ACT35 | FITC | 1:25 | BD Pharmingen |
| ICOS (mouse) | human | G44-26 | PE | 1:25 | BD Pharmingen |
| 41BB (mouse) | human | 4B4-1 | PerCP-Cy5.5 | 1:100 | BioLegend |
